# DNA Supercoiling, Topoisomerases, and Cohesin: Partners in Regulating Chromatin Architecture?

**DOI:** 10.3390/ijms19030884

**Published:** 2018-03-16

**Authors:** Camilla Björkegren, Laura Baranello

**Affiliations:** 1 Department of Cell and Molecular Biology, Karolinska Institutet, 17177 Stockholm, Sweden; Camilla.Bjorkegren@ki.se; 2 Department of Biosciences and Nutrition, Karolinska Institutet, 141 43 Huddinge, Sweden

**Keywords:** DNA topology, transcription, cohesin, CTCF, genome organization, topoisomerase

## Abstract

Although our knowledge of chromatin organization has advanced significantly in recent years, much about the relationships between different features of genome architecture is still unknown. Folding of mammalian genomes into spatial domains is thought to depend on architectural proteins, other DNA-binding proteins, and different forms of RNA. In addition, emerging evidence points towards the possibility that the three-dimensional organisation of the genome is controlled by DNA topology. In this scenario, cohesin, CCCTC-binding factor (CTCF), transcription, DNA supercoiling, and topoisomerases are integrated to dictate different layers of genome organization, and the contribution of all four to gene control is an important direction of future studies. In this perspective, we review recent studies that give new insight on how DNA supercoiling shape chromatin structure.

## 1. Hierarchy and Principles of Genome Organization 

The three-dimensional structure of the genome is essential to dictate its biological function. Within the nucleus, the linear sequence of DNA is spatially arranged in a defined manner that allows effective control of gene expression. Transcriptional output depends on a complex interplay between proteins acting within the promoter region of genes and through long range chromosomal interactions. Gene regulation by chromosomal interactions has become evident in the most recent years, thanks to advances in imaging and chromosome conformation capture (3C)-based techniques, which have revealed how chromosomes are folded at different scales. Even though these observations have started to provide answers regarding how gene regulation is achieved in the dense chromatin environment of the nucleus, the mechanisms and factors orchestrating chromosome organization remain unclear.

**Figure 1:**
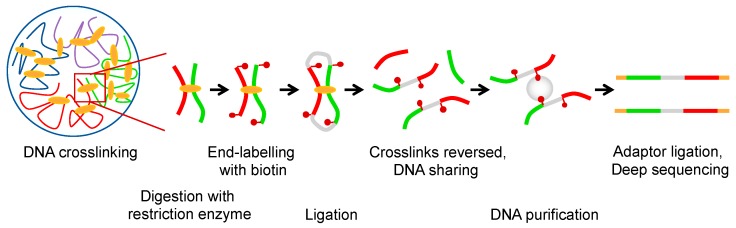
Whole-genome chromatin conformation capture (Hi-C) detect chromosomal rearrangements. Overview of the Hi-C method. Briefly, cells are first treated with formaldehyde, which crosslinks chromatin segments that are spatially adjacent. The crosslinked chromatin is thereafter digested with restriction enzyme(s). The resulting DNA fragments are labelled with biotinylated nucleotides and subjected to ligation. Crosslinks are reversed, DNA sheared, and biotinylated fragments are isolated using streptavidin beads. Finally, deep sequencing of the purified fragments identifies which chromosomal regions were in proximity at the time of formaldehyde cross-linking. Recently, the resolution of Hi-C has been improved by replacing restriction enzyme cleavage with micrococcal nuclease (MNase) digestion [23], or using in situ Hi-C, in which DNA-DNA proximity ligation is performed in intact nuclei [24].

**Figure 2:**
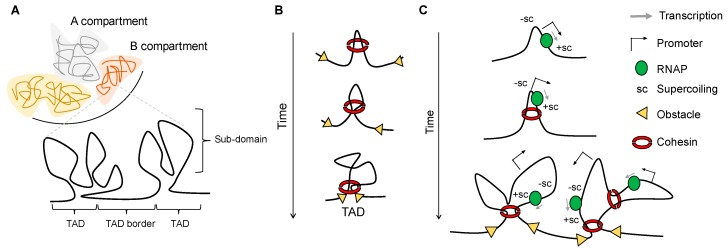
The 3D genome and factors contributing to its organization. (**A**) In the nucleus interphase, chromosomes are organized in A/B compartments, which in turn contain topologically associating domains (TADs) and sub-TADs; (**B**) Models for domain formation by cohesin as a loop extruding factor; (**C**) Transcription-generated supercoiling can contribute to TADs structure. Positive supercoiling generated ahead of the moving RNA polymerase (RNAP) could promote cohesin sliding along the DNA until the complex meets an obstacle such as CTCF or, possibly, another region of supercoiled DNA. Negative supercoiling favours loop formation and stabilization of promoter-enhancer interactions.

Interphase chromosomes are compartmentalized, and the spatial positioning of genomic segments affects ongoing DNA transactions. The first whole-genome chromatin conformation capture (Hi-C, Figure 1) experiments [1] provided low-resolution contact maps (1 Mb) [2]. This revealed a checkerboard pattern of interactions, which reflects spatial segregation of transcriptionally active (A compartment) and inactive (B compartment) chromatin. Contacts between genomic regions belonging to the same compartment are more frequent than between regions of different ones [2]. With increasing Hi-C resolution (50 kb) (Figure 1), a second layer of hierarchical organization was discovered. Chromosomes are folded into topologically associating domains (TADs), which in mammals are 200 kb to 1 Mb regions within which the likelihood of DNA-DNA interactions is increased as compared to regions outside the particular TAD. The size of TADs is limited by boundaries that are enriched in highly expressed genes [3] (Figure 2A). TADs are thought to contribute to gene expression by favouring or preventing promoter-enhancer interactions and have been linked to fundamental biological processes such as genomic imprinting [4] and developmentally regulated programs [5]. Since TADs of varying size have been found in many other organisms, including bacteria, yeasts, and plants [6], it seems that the organization of chromosome into self-interacting domains is a unifying principle in chromosome biology. Within TADs, loops or sub-domains with higher contact frequencies are formed and are visible as focal enrichments in contact frequencies between paired loci in Hi-C maps (Figure 2A). In addition, sub-domains are also found within TADs boundaries. It is not clear whether these domains are different from TADs or if they simply represent a further level of hierarchical organization [7]. However, it is widely accepted that architectural proteins such as CCCTC-binding factor (CTCF) and cohesin play a major role in TAD formation, their stabilization, and intra-domain organization [8]. CTCF is a highly conserved protein with a unique structure that confers a versatile role in genome function. By binding to specific sequences [9], CTCF has been implicated in promoter repression, enhancer insulation, and chromatin loop stabilization [10]. Cohesin is a conserved ring-shaped protein complex thought to topologically enclose chromatid fibres in a sequence-independent manner [11,12]. This complex mediates cohesion between sister chromatids [13,14] and regulates gene expression, the latter likely through its role in forming chromatin loops [15,16,17]. Even though often co-localized at the base of loops [18], CTCF and cohesin seem to have distinct roles in shaping chromatin architecture [19]. According to the loop extrusion model, one of the most attractive and elegant hypotheses to describe the mechanism of loop formation in interphase chromosomes [7,20,21,22], a loop is generated by extrusion through one or two cohesin rings. The extrusion stops when these rings meet an obstacle, for example, a DNA site occupied by CTCF on each side of the growing loop (Figure 2B).

How does cohesin travel along the chromatin fibre extruding the DNA through its ring? Although cohesin is an ATPase, and the cohesin-related complex condensin has been shown to create DNA loops and translocate along DNA in an ATP-dependent manner in vitro [25,26], there is as of yet no evidence that cohesin has an intrinsic ability to move along DNA. However, early characterization of cohesin binding pattern in the budding yeast genome indicated that transcription localizes cohesin at intergenic regions between convergently oriented genes [27]. This suggests that the RNA polymerase (RNAP) pushes cohesin along chromosomes, and using a combination of genomic and genetic approaches, Peters and colleagues recently showed that in the absence of CTCF and Wapl—a cohesin dissociating factor—human cohesin also redistributes to sites of convergent transcription. Moreover, if the rate of transcription of the converging genes differs, cohesin is positioned asymmetrically in such a manner that suggests that transcription of the more active genes pushes cohesin into the more weakly expressed gene [28]. This seems to indicate that the transcription machinery can relocate cohesin over long distances in the eukaryotic genome. Additionally, Mizugichi et al. have shown that TADs in *S. pombe* are oriented in a way that transcription direction points outwards from the centre of the domain so that the boundaries are flanked by genes in a convergent orientation [27].

The transcription-dependent sliding of cohesin along chromatin could be caused by sterical/architectural constrains due to the sizes of the polymerase and cohesin complexes. Alternatively, the torque introduced into DNA by the moving RNAP could be another determinant of cohesin re-localization. The transcribing RNAP induces axial rotation in the transcribed DNA, which should promote polymerase rotation relative to the DNA. However, the rotation of RNAP is hindered due to the viscous drag or the tethering to nuclear structures. Due to this, transcription will change the structure of the DNA helix, causing it to become over-twisted (positively supercoiled) in front of the polymerase, and under-twisted (negatively supercoiled) behind the polymerase [29,30]. Topoisomerases (Top1 and Top2) drain supercoils by transiently breaking and resealing DNA strands [31]. In vitro transcription experiments and single molecule analysis have proven that even at high concentration, topoisomerases are unable to fully release this superhelical stress introduced by RNAP [32,33]. Thus, a certain level of supercoiling will remain stored in the DNA. This supercoiling has the potential to affect protein-DNA interactions, as it was shown for nucleosomes, which are displaced ahead of transcribing RNAPII [34,35], and for the cohesin-like protein Smc5/6, the DNA association of which appears to be controlled by torsional stress [36,37]. Therefore, it is tempting to hypothesize that the positive supercoiling generated by RNAP, instead of the transcription machinery in itself, could “push” cohesin along the double helix, providing an impulse for the extrusion of the loop (Figure 2C).

In addition to this, supercoiling could also constitute a barrier to the movement of proteins traveling along the helix, as suggested by the observation of polymerase stalling in response to supercoil accumulation [38]. This happens because the more intensely a gene is transcribed, the more intense the opposing torsional resistance to translocation becomes. At highly transcribed or convergently oriented genes the supercoiling may accumulate and surmount levels that stall the polymerase. The same principle can be applied to other factors moving together with RNAP, such as cohesin: supercoiling could become an obstacle to the moving of a sliding cohesin, which would be forced to arrest, defining an anchor point for the growing loop. In this instance, supercoiling could potentially exert the same function described for CTCF in loop extrusion. This could explain the accumulation of cohesin at sites of convergent transcription, and how TADs form in organisms lacking CTCF. It is plausible that during evolution multiple mechanisms have evolved independently to contribute to domain formations of different sizes and persistence.

The ability to read out the topology of the genome is progressing fast, but the contribution of DNA topology or the role of DNA relaxing enzymes to genome organization has received less attention. Below, we will discuss recent experimental studies and dynamic simulations showing how DNA topology might alter the properties of the chromatin fibre, thus shaping nuclear architecture.

## 2. Transcription-Generated Supercoiling as a Driving Force for Domain Organization

**Figure 3:**
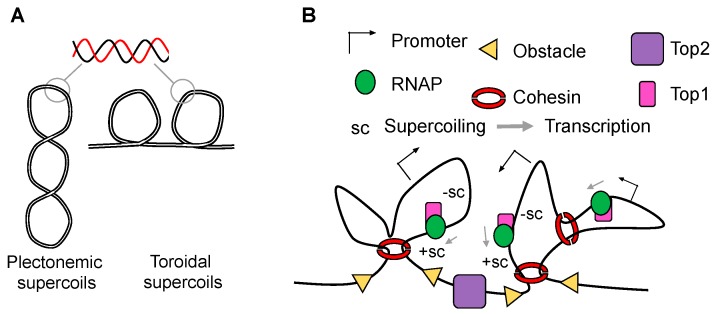
Supercoiling contributes to loop formation. (**A**) Supercoiled DNA folds into plectonemic (left) or toroidal (right) conformations. While plectonemic structures have been observed in bacterial chromosomes, toroidal supercoils are found constrained within nucleosomes. Here, both cartoons depict negative supercoils. For a detailed description of DNA topology, please see [53]; (**B**) Topoisomerase activity is regulated to ensure that negative supercoiling is maintained along transcribed units. Positive supercoils accumulate in front of the polymerase.

Supercoiling is a fundamental property of DNA and can assume toroidal or plectonemic configurations (Figure 3A). It is generated by molecular machineries such as polymerases, as a consequence of DNA strand separation, and chromatin remodelling complexes due to their nucleosome displacement activities [29]. Supercoiling can exist in a constrained state, within nucleosome complexes, or an unconstrained state, free to dissipate along the helix. Despite its key role in genome regulation [39], it has proved difficult to study DNA supercoiling in the eukaryotic genome because of its dynamic nature, and due to the lack of experimental approaches to map supercoiling in vivo. However, recent advances have enabled a switch from mostly theoretical descriptions of DNA supercoiling and in vitro studies to functional studies in vivo. Psoralen-based assays [40] combined with genomic approaches have led several groups to provide an unprecedented view of DNA topology in living cells [35,41,42,43]. Based on the work by Levens and Kouzine [42], we know that dynamic supercoiling is a feature of virtually every transcribed promoter, and can spread approximately 2.0 kb upstream of transcription start sites [42]. Top1 and Top2 are differentially recruited and distinctly deployed at differentially expressed genes to establish a robust level of negative supercoiling within promoter areas. These finding support a model where rates of transcription and DNA relaxation are coordinated, and the regulated topoisomerase activity not only is essential to remove topological obstacles to transcription but also to preserve negative supercoiling within gene regulatory regions [44]. The same group has also recently demonstrated that the transcription machinery actively manages Top1 relaxation to regulate the removal of torsional stress and favour transcription [45]. Top1 bound to promoters is relatively inactive and only becomes fully active as it progresses into the body of the gene. When the carboxyl-terminal domain of RNAPII is progressively phosphorylated by the kinase Brd4, it stimulates Top1 activity above its intrinsic relaxation state. Therefore, even if the net state of the genome is torsionally relaxed [46], the transcriptional machinery actively maintains negative DNA supercoils in localized regions [42] to alter the chromatin fibre and contribute to transcriptional regulation [47]. Unconstrained DNA (found between nucleosomes) partially unwinds in response to negative supercoiling, whereas nucleosomal DNA behaves differently. As DNA is tightly associated to nucleosomes, untwisting of DNA causes chromatin fibres to rotate, enabling the superhelical turns to be transmitted throughout the genome. Although there is no direct evidence for the transmission of torque through chromatin, the phenomenon could explain the dissipation of supercoiling near the ends of yeast chromosome [41] and also explain why supercoiling accumulation is more problematic for large chromosomes whereas its dissipation is less achievable through chromosome rotation [36]. Using magnetic tweezers to introduce torsional stress into nucleosome arrays, Bancaud et al. also found that chromatin can accommodate a certain level of supercoiling [48]. Therefore, within a topological domain, the chromatin fibre is expected to adsorb a certain amount of supercoiling but eventually it becomes distorted and folds into a supercoil. As a consequence, any two sites in the same topological domain are on average closer to each other. [49]. In line with this, by including supercoiling into in silico models of topological domain organization one can reproduce several experimentally observed characteristics of interphase chromosomes, such as their contact maps [50]. Supercoiling can cause formation of TADs, i.e., regions with a 2- to 3-fold increased frequency of contacts as compared to loci with similar genomic distances but located in different TADs [50]. The increased contact interactions could in turn enhance the fraction of time during which enhancers and promoters stay together [51]. Enhancer-promoter communication is one of the major rate-limiting steps during eukaryotic transcription, and supercoiling regulation might represent an effective and fast way to achieve new and persistent interactions [52].

RNA-sequencing experiments have shown that many enhancers are transcribed. Topoisomerases have been found to be associated to enhancers where they promote transcriptional activation through regulatory interactions with enhancer specific factors, and by stimulating production of RNA transcripts (eRNAs) derived from enhancer sites [54,55]. The function of eRNAs is still unknown, but significant correlations have been observed between gene expression, promoter-enhancer interactions, and the presence of enhancer RNAs [56]. Interestingly, the transcription of eRNAs precedes the transcription of genes they regulate [57]. Therefore, the DNA supercoiling emanating from these regions might be responsible for altering the local chromatin environment in a manner which favours efficient sliding of chromatin fibres, thereby bringing enhancers and promoters into close contact. Lastly, the disassembly of nucleosomes to favour promoter and enhancer accessibility to transcription factors (achieved by chromatin remodellers) also generates negative supercoiling. Therefore, nucleosome displacement could, together with enhancer transcription, introduce the initial wave of supercoiling needed for TADs formation and/or stabilization.

One implication of this hypothesis is that negative supercoiling and active transcription would be expected to correlate. In this regard, it has been reported that DNA supercoiling is organized into large domains of about 100 kb in size with specific topological features: those with a negatively supercoiled, transcription permissive environment, and those that are positively supercoiled, more compacted, and more likely to be silent [43]. These supercoiling domains are formed and remodelled by RNAPII and topoisomerases activity and are flanked by CTCF binding sites. Partially overlapping with TADs, their structure varies depending on the transcriptional environment, indicating that supercoiling domains are dynamic properties of the chromatin fibre rather than static structural features. Top1 is enriched along the more negatively supercoiled domain, while Top2, which shows significant affinity for regions with juxtaposition of DNA duplexes [58], has been mostly found at loop bases in combination with CTCF and cohesin [18,59]. At these sites, Top2-associated double strand breaks can be detected [60]. Top2 has also been found synergizing with chromatin remodelling complexes where it might disentangle the DNA in the transition between facultative heterochromatin to accessible chromatin [61]. Whether these two Top2 functions are part of the same process is still unclear, but it is tempting to speculate that Top2, together with chromatin remodelling complexes, might act at loop boundaries in a manner that helps to promote the switch from a compacted heterochromatic state to an accessible state [61]. These observations invite the inclusion of topoisomerases into models of topological domain regulation as follows [62,63]. By relaxing positive supercoiling [64] at the base of the loop, Top2 will mostly contribute to its stabilization and isolation. On the other hand, controlled Top1 activity in combination with transcription within the loop will ensure that a sturdy level of negative supercoiling will distort the geometry of DNA and change its properties to promote chromatin interactions (Figure 3B).

## 3. Independent Layers of Chromatin Organization

Even though in mammals architectural proteins such as CTCF and cohesin seem to have a key role in high-order chromatin organization, emerging evidence suggests that in lower eukaryotes, prokaryotes, and plants, all of which lack CTCF, transcriptional activity and DNA topology are likely to play a similar role in establishing TAD-like domains. Topological domains could be transcription- and topoisomerases-dependent and composed of supercoiled regions forming plectonemes. This is the case of the bacterial genome, which is organized in chromosomal interaction domains (CIDs) determined by supercoiling and transcription, whose structures and features resemble eukaryotic TADs [65]. Modelling studies suggested that individual CIDs are composed of supercoiled regions forming plectonemes, and that the borders between topological domains are plectoneme-free. Contact domains reminiscent of those seen in mammals have been identified also in *C. elegans* and *A. thaliana*, which have no known CTCF homologs [6]. The mechanisms responsible for the establishment of contact domains in these organisms are still unknown, but we propose that the transcriptional state is a major predictor of Hi-C contact maps. In these organisms, domain boundaries appear at transcriptionally inactive–active switches, and the size of a domain depends on the length of contiguous active or inactive genomic regions.

TADs have also been identified in *Drosophila*, but CTCF does not have a role in TAD formation. Applying Hi-C and Hi-ChIP (a chromatin conformation-based method coupled to antibody immunoprecipitation [66]) to the *Drosophila* genome, Rowley et al. found no evidence of looping mediated by CTCF at the borders of TADs [67]. Even though they could not exclude the possibility that other architectural proteins might work at TAD boundaries [68], their observation indicated that TADs are a direct result of the establishment of A/B compartments defined by the chromatin state of their interior (active or repressed) rather than by a border element. Finally, even though the loss of cohesin in mammalian cells leads to a severe depletion of TADs [69,70], A/B compartments are maintained, suggesting that TADs and compartments are formed by independent mechanisms.

By increasing contact frequency between distal regions of the chromatin fibre, gene activation and transcription-generated supercoils could promote formation of the A compartment, which will in turn exclude heterochromatic repressed regions forming the B compartment. It is also possible that other types of factors would drive the physical interactions between genes in the same transcriptional state. Clustering of proteins and phase separation events may play a role in the establishment of compartmental domains, as recently described for the formation of super-enhancers [71] and heterochromatin domains [72]. Super-enhancers are clusters of constitutive enhancers spanning long regions of the genome and characterized by an unusually high density of DNA binding proteins [73]. Several lines of evidence indicate that the formation and function of super-enhancers involve cooperative processes that bring many constitutive enhancers into close proximity. High densities of proteins have been implicated in the formation of membraneless organelles, which are formed by phase separation events that play key roles in compartmentalizing biochemical reactions within cells [71,74]. Phase-separation has also been observed to be involved in the formation of heterochromatin, as the *Drosophila* protein HP1a nucleates into foci that display liquid properties during the formation of heterochromatin domains [72]. Overall, this suggests that multivalent macromolecular interactions and phase separation events could be major driving forces in nuclear compartmentalization [75], thereby contributing to the formation of membraneless A/B compartments [76]. It is plausible that many of these macromolecular interactions will not form without strong bending of DNA. Supercoiling has the ability to increase the flexibility of DNA [77] and would therefore also favour multiple contacts between molecular partners [78].

Altogether, this suggests that in organisms that lack architectural proteins such as CTCF, the transcriptional state of chromatin is a critical factor in the formation of compartmental domains, while the organization of mammalian chromosomes is established via a combination of such compartmental domains and point-to-point CTCF-cohesin interactions. In other words, compartmental domains represent the primary mechanism underlying 3D chromatin organization, and architectural proteins are responsible for additional interactions that establish the complex architecture of the mammalian nucleus.

## 4. Conclusions

Understanding how the genome is organized has become a topic of intense interest and an increasingly growing number of studies, and analyses of the interplay between interphase chromatin and architectural proteins have revealed key features of the 3D structure. Nevertheless, in recent years it has become evident that the structure of the DNA helix in itself is also part of the regulation. By modulating the dynamic rearrangement of the chromatin fibre, DNA supercoiling can control a DNA-based process across long distances. This observation invites the consideration of supercoiling and topoisomerases as active players in domain organization. Our understanding of this phenomenon is still elusive, and improvements of techniques to map DNA supercoiling, topoisomerase activity, and chromatin looping, and their integration with the study of phase-separated systems, are needed to eventually clarify the interrelationships between DNA topology, chromatin topology, and genome function.
